# Predicting Hemodynamic Fluctuations During Adrenalectomy for Pheochromocytoma

**DOI:** 10.3390/diagnostics16020340

**Published:** 2026-01-21

**Authors:** Marina Stojanovic, Magdalena Grujanic, Anka Toskovic, Milan Jovanovic, Biljana Milicic, Matija Buzejic, Branislav Rovcanin, Boban Stepanovic, Vladan Zivaljevic

**Affiliations:** 1 Faculty of Medicine, University of Belgrade, 11000 Belgrade, Serbia; marinailicstojanovic@gmail.com (M.S.); milanjovanovicceh@gmail.com (M.J.);; 2 Center for Anesthesiology and Resuscitation, Clinical Center of Serbia, 11000 Belgrade, Serbia; 3 Clinic for Endocrine Surgery, University Clinical Centre of Serbia, 11000 Belgrade, Serbia; 4 Faculty of Dental Medicine, University of Belgrade, 11000 Belgrade, Serbia

**Keywords:** pheochromocytoma, adrenalectomy, hemodynamic instability, metanephrines, normetanephrines

## Abstract

**Background:** Pheochromocytoma is a rare adrenal neuroendocrine tumor characterized by excessive catecholamine secretion, which can lead to significant perioperative hemodynamic instability. Despite advances in anesthetic and surgical management, intraoperative hypotension is a common complication. This study aimed to identify preoperative and intraoperative predictors of hemodynamic instability during adrenalectomy for pheochromocytoma in order to improve intraoperative management and patient safety. **Methods:** This retrospective study included adult patients who underwent adrenalectomy for pheochromocytoma at the University Clinical Center of Serbia between January 2022 and June 2025. Preoperative clinical and biochemical data, tumor characteristics evaluated by imaging methods (CT or MRI), surgical approach, and intraoperative hemodynamic parameters were analyzed. Intraoperative hypotension was defined as mean arterial pressure (MAP) < 60 mmHg despite adequate volume resuscitation. Univariate and multivariate logistic regression analyses were performed to identify predictors of hypotension. **Results:** A total of 51 adult patients were included in the analysis. Intraoperative hypotension occurred in 26 patients (51%) and was significantly associated with larger tumor size and increased intraoperative fluid requirements. Multivariate analysis identified tumor diameter ≥ 49 mm (OR 0.176, 95% CI 0.034–0.895, *p* = 0.036) and intraoperative crystalloid infusion ≥ 1200 mL/h (OR 0.132, 95% CI 0.030–0.574, *p* = 0.007) as independent predictors of intraoperative hypotension. Preoperative catecholamine levels, surgical approach, and type of alpha-blocker were not significant predictors. **Conclusions:** Tumor size was identified as a significant predictor of intraoperative hemodynamic instability during adrenalectomy for pheochromocytoma. Careful preoperative assessment and individualized intraoperative fluid management may help reduce the risk of hypotension and optimize perioperative outcomes.

## 1. Introduction

Pheochromocytoma is a rare neuroendocrine tumor that develops from catecholamine-producing chromaffin cells of the adrenal medulla, with an incidence of 0.8 per 100,000 patients per year [1].

Adrenalectomy remains the only curative treatment for pheochromocytoma, regardless of whether the tumor is benign or malignant. Although approximately 90% of tumors are benign, perioperative hemodynamic instability remains a significant factor in morbidity and mortality. Chronic catecholamine excess, along with daily physiological triggers, may predispose patients to cardiovascular events even before surgery, thereby increasing perioperative risk [2,3]. Preoperative management commonly includes alpha- and beta-adrenergic blockade, intravascular volume expansion to counteract chronic catecholamine-induced vasoconstriction, and careful intraoperative hemodynamic monitoring, all aimed at reducing perioperative complications [4,5].

Manipulation of the tumor during surgery triggers additional catecholamine release, inducing hypertensive crises and a range of cardiac rhythm disturbances, including tachyarrhythmias, bradyarrhythmias, atrial fibrillation, and ventricular ectopy, which can compromise cardiac output and increase perioperative cardiovascular risk [2,3].

After tumor resection and ligation of the adrenal vein, the abrupt drop in circulating catecholamines can lead to loss of vasoconstrictor tone and the development of hypotension, which may persist for up to 48 h. Early identification and appropriate management of hypotension during the intraoperative period are essential to prevent complications arising from acute organ hypoperfusion [1,6]. Ruan et al. [7] demonstrated that prolonged exposure to mean arterial pressure (MAP) values below 85, 80, 75, 70, or 65 mmHg significantly increases the risk of acute kidney injury, with the duration of hypotension playing a critical role. Moreover, myocardial perfusion may be compromised during surgery due to dynamic and pronounced blood pressure fluctuations, compounded by prior catecholamine-induced toxicity affecting the coronary vessels and myocardium, which can lead to structural and functional cardiac remodeling [8,9].

Numerous studies have identified multiple potential predictors of intraoperative hypotension in patients with pheochromocytoma. The most commonly reported factors include elevated preoperative catecholamine levels [10], tumor size [11,12,13], choice of surgical approach [14], and the type and duration of preoperative alpha-blockade [15,16,17]. Traditionally, intravascular volume expansion has been recommended to attenuate intraoperative hemodynamic fluctuations resulting from chronic catecholamine-induced vasoconstriction [18]. However, recent evidence suggests that alpha-blockade without preoperative volume expansion does not increase the risk of intraoperative hypotension [19,20], while Liu et al. [21] have reported that excessive preoperative fluid loading may adversely affect hemodynamic stability.

The aim of this study was therefore to determine the incidence of intraoperative hypotension in patients with pheochromocytoma and to identify key predictors of its occurrence, with the goal of improving strategies for optimal hemodynamic control and reducing the risk of adverse outcomes.

## 2. Material and Methods

### 2.1. Study Design and Patient Population

This retrospective study included patients who underwent surgical treatment for pheochromocytoma at the Clinic for Endocrine Surgery, University Clinical Center of Serbia, between January 2022 and June 2025. The study included patients older than 18 years who underwent preoperative functional evaluation of adrenal gland activity.

Inclusion criteria required elevated levels of urinary or plasma epinephrine, norepinephrine and their metabolites (metanephrines and normetanephrines), as determined by standard preoperative biochemical assessment. Hormonal evaluation was performed as part of routine diagnostic work-up at the Department of Endocrinology, with hormone levels interpreted according to laboratory reference ranges; Blood and urine samples were collected at variable times based on clinical circumstances. For urinary catecholamine measurements, 24 h urine collections were conducted in accordance with standard clinical protocols, starting from the second morning void and continuing for the subsequent 24 h, including the first morning void of the following day.

The diagnostic evaluation of pheochromocytoma and initial medical optimization for patients with positive biochemical findings were completed in the Department of Endocrinology. Following appropriate endocrine assessment and preparation, patients were referred to the Department of Endocrine Surgery for anesthesiological evaluation and surgical treatment. Tumor localization and growth were assessed using MRI and/or CT imaging. Patients without complete biochemical, imaging, or intraoperative data were excluded from the analysis. Histopathological confirmation of pheochromocytoma following adrenalectomy was required for inclusion in the final analysis.

The study protocol was approved by the Ethics Committee of the University Clinical Center of Serbia, No 1787/32, Date: 27 November 2025. Written informed consent for surgical treatment and the use of anonymized clinical data for research purposes was obtained from all patients.

### 2.2. Preoperative Management and Data Collection

Preoperative demographic and clinical data were collected from medical records and the electronic database (Heliant) of the Clinic for Endocrine Surgery, including sex, age, tumor size and localization, catecholamine levels and their metabolites, maximal systolic and diastolic blood pressure, type of alpha-blockade, and planned surgical approach (laparoscopic retroperitoneal, lateral, or open).

Standardized preoperative pharmacological preparation included alpha-blocker therapy of 7–28 days, adjusted according to individual patient response. Symptomatic tachycardia was managed with a selective beta-1 blocker added at least two days prior to surgery. Once stable blood pressure (<160/100 mmHg) and heart rate (<80 bpm) were achieved and maintained, patients were transferred to the surgical ward. Alpha-blockers were discontinued 12–36 h prior to surgery, along with fluid administration of at least 1500 mL/day. If hypertension occurred following alpha-blocker discontinuation and before the surgical intervention, short-acting antihypertensive agents were administered as needed.

### 2.3. Anesthetic and Surgical Technique

Before induction of general endotracheal anesthesia, invasive arterial blood pressure monitoring was established in all patients. Standard monitoring included electrocardiography, pulse oximetry, capnography, and Bispectral Index (BIS). General anesthesia was administered according to standard institutional practice and induced using fentanyl (1–2 μg/kg), propofol (2 mg/kg), and rocuronium bromide (0.6 mg/kg). Central venous access was obtained when clinically indicated, and nasogastric and urinary catheters were placed after induction. Anesthesia was maintained with sevoflurane to ensure an adequate depth of anesthesia (BIS 40–60). Vasoactive agents were administered as required to maintain intraoperative hemodynamic stability.

Adrenalectomy was performed using a laparoscopic, retroperitoneoscopic or open approach, according to tumor characteristics, patient-related factors, and surgeon preference. Tumor size was defined as the maximum diameter measured on preoperative CT/MRI imaging.

### 2.4. Intraoperative Monitoring and Data Collection

During the intraoperative period, the following parameters were recorded: duration of anesthesia and surgery (in minutes), lowest recorded systolic and diastolic blood pressure values, intraoperative fluid administration, use of vasopressors and vasodilators. Hemodynamic parameters were continuously monitored throughout the procedure. Particular attention was paid to hypotensive episodes occurring after tumor resection, a period commonly associated with significant hemodynamic changes.

### 2.5. Management of Intraoperative Hemodynamic Instability

Hypertensive crises (BP > 180/100 mmHg) were managed with nitroglycerin, either as monotherapy or in combination with urapidil. Following tumor resection, hypotension was initially treated with isotonic crystalloid fluid replacement according to a standardized institutional protocol, aiming to maintain adequate intravascular volume. A minimum total fluid resuscitation of 25 mL/kg body weight was administered, including 1000 mL preoperatively and an additional 1000–1500 mL as a rapid intravenous infusion, with adjustments made based on continuous invasive arterial blood pressure monitoring and the patient’s clinical status.

In cases of refractory hypotension, defined as mean arterial pressure (MAP) < 60 mmHg despite adequate fluid resuscitation, norepinephrine was administered to maintain a target MAP > 65 mmHg. The duration and rate of norepinephrine infusion were individualized according to the patient’s hemodynamic response, and in some cases, support continued into the early postoperative period.

Patients were categorized into two groups based on the presence or absence of intraoperative hypotension. Tumor size was defined as the largest diameter measured on CT/MRI imaging, while tumor volume and additional imaging characteristics (Hounsfield units, necrosis, or vascularization) were not evaluated. Urinary catecholamine levels were expressed in nmol/24 h and their metabolites in µmol/24 h, with additional categorization according to whether values were below or exceeded a fivefold increase above the laboratory reference range. Following histopathological confirmation, the PASS score (Pheochromocytoma of the Adrenal Gland Scaled Score) of the resected tumor was determined. The PASS score was evaluated both as a continuous variable and as a categorical variable (<4 vs. ≥4). The associations of catecholamine levels, PASS score, and other relevant clinical variables with the occurrence of intraoperative hypotension were subsequently analyzed.

### 2.6. Statistical Methods

Statistical analyses were performed using IBM SPSS Statistics, version 25 (IBM Corp., Armonk, NY, USA). The normality of continuous variables was assessed using the Kolmogorov–Smirnov test and visual inspection of histograms. Continuous variables were compared between groups using the independent *t*-test for normally distributed data and the Mann–Whitney U-test for non-normally distributed data. Categorical variables were compared using the chi-square test or Fisher’s exact test, as appropriate.

Univariate logistic regression was performed to identify variables associated with intraoperative hypotension. Variables with a *p*-value < 0.1 in univariate analysis were subsequently included in the multivariate logistic regression model to determine independent predictors, with odds ratios (OR) and 95% confidence intervals (CI) reported. Model fit was assessed using the Nagelkerke R^2^. All tests were two-tailed, with statistical significance set at *p* ≤ 0.05.

## 3. Results

### Patient Characteristics

A total of 51 patients were included in the study, of whom 60.8% were female and 39.2% male, with a mean age of 53 years (range 20–79). The mean tumor diameter was 49.04 ± 29.41 mm, with the largest tumor measuring 144 mm in diameter. Tumors were evenly distributed between the left (45.1%) and right (54.9%) adrenal glands. Laparoscopic adrenalectomy was the most frequently performed surgical approach (54.9%), followed by retroperitoneoscopic adrenalectomy (29.4%), while open adrenalectomy was performed in 15.7% of cases.

Table 1 summarizes the mean 24 h urinary catecholamine levels and their metabolites, which demonstrated considerable variability (see Table 1). Preoperative blood pressure measured before alpha-blocker initiation was elevated, with a mean systolic blood pressure of 181.3 ± 35.4 mmHg and diastolic blood pressure of 101.1 ± 20.1 mmHg. Regarding preoperative pharmacological preparation, 43.1% of patients received phenoxybenzamine, 47.1% doxazosin, and 9.8% did not receive any alpha-blocker therapy. The duration of alpha-blocker therapy ranged from 7 to 28 days, adjusted according to clinical response.

According to the predefined criteria, intraoperative hypotension occurred in 26 (51%) of the 51 patients. Table 2 presents the comparison of preoperative characteristics between patients who developed intraoperative hypotension and those who did not. Patients with intraoperative hypotension had a significantly larger tumor diameter compared to those without hypotension (59.88 ± 36.43 mm vs. 37.76 ± 12.60 mm, *p* = 0.037). No significant differences were observed between the groups regarding sex, age, or tumor laterality. The PASS score was higher in patients with intraoperative hypotension (mean ± SD: 4.12 ± 2.12) compared to patients without hypotension (3.08 ± 1.72), showing a trend toward significance, but this did not reach statistical significance (*p* = 0.094).

Preoperative 24 h urinary catecholamine and metabolite levels did not differ significantly between groups, nor did the maximum systolic and diastolic blood pressures recorded during hypertensive episodes before the initiation of alpha-blocker therapy. Although doxazosin use was more frequent in the hypotension group, this difference did not reach statistical significance regarding the type of preoperative pharmacological preparation.

**Table 2:** Patients and tumor characteristics according to the occurrence of intraoperative hypotension.

	Intraoperative Hypotension (−)	Intraoperative Hypotension (+)	*p*-Value
Variables	*N* = 25 (49%)	*N* = 26 (51%)	
Age (years, mean ± SD)	50.40 ± 15.04	55.65 ± 12.89	0.186
Sex Female Male	15 (60.0%) 10 (40.0%)	16 (61.5%) 10 (38.5%)	0.910
Smoking Smoker Non–smoker	10 (45.5%) 12 (54.5%)	9 (39.1%) 14 (60.9%)	0.668
Tumor diameter (mm, mean ± SD)	37.76 ± 12.60	59.88 ± 36.43	0.037 *
Side of PHEO Left-sided Right-sided	12 (48.0%) 13 (52.0%)	11 (42.3%) 15 (57.7%)	0.683
PASS score <4 ≥4	15 (60%) 9 (36%)	15 (57.7%) 11 (42.3%)	0.094
24 h urinary norepinephrine (nmol/24 h)	2121.85 ± 3695.02	2019.18 ± 3944.64	0.972
24 h urinary epinephrine (nmol/24 h)	381.12 ± 596.04	293.40 ± 380.43	0.570
24 h urinary dopamine (nmol/24 h)	2899.61 ± 2684.39	2323.98 ± 1288.07	0.782
24 h urinary metanephrine (µmol/24 h)	20.42 ± 38.33	24.41 ± 61.42	0.660
24 h urinary normetanephrine (µmol/24 h)	35.26 ± 34.94	34.94 ± 104.75	0.266
Preoperative SBP (before alpha-blocker) (mmHg, mean ± SD)	174.72 ± 36.98	187.54 ± 33.31	0.132
Preoperative DBP (before alpha-blocker) (mmHg, mean ± SD)	101.96 ± 22.05	100.27 ± 18.38	0.985
Preoperative Alpha blocker administration Phenoxybenzamine Doxazosin None	12 (48.0%) 11 (44.0%) 2 (8.0%)	10 (38.5%) 13 (50.0%) 3 (11.5%)	0.768

* Statistically significant (*p* < 0.05). Data are presented as mean ± SD or n (%). PHEO, pheochromocytoma; SBP, systolic blood pressure; DBP, diastolic blood pressure; PASS, Pheochromocytoma of the Adrenal Gland Scaled Score. Group comparisons were performed using Student’s *t*-test or Mann–Whitney U test for continuous variables, and chi-square or Fisher’s exact test for categorical variables.

Table 3 presents perioperative variables stratified by the presence of intraoperative hypotension. There were no significant differences in the duration of surgery or anesthesia between patients with and without hypotension (surgery: 93.46 ± 30.35 vs. 92.00 ± 38.19 min, *p* = 0.720; anesthesia: 125.00 ± 33.55 vs. 121.60 ± 42.05 min, *p* = 0.597). The type of surgical approach did not differ significantly between groups (*p* = 0.334).

Intraoperative crystalloid infusion was significantly higher in the hypotension group (median 2000 [1000–3500] mL vs. 1500 [500–3000] mL, *p* < 0.001).

The use of antihypertensive agents intraoperatively showed a trend towards higher utilization in the hypotension group, but did not reach statistical significance (*p* = 0.054). Additionally, the requirement for vasopressor support during surgery was significantly more frequent in the hypotension group (50% vs. 0%, *p* < 0.001), whereas the remaining 13 patients with hypotension were successfully managed with fluid replacement alone. The administration of diuretics, length of hospital stay, and incidence of postoperative hypotension did not differ significantly between groups.

**Table 3:** Perioperative variables according to the occurrence of intraoperative hypotension.

Characteristics	Intraoperative Hypotension (−)	Intraoperative Hypotension (+)	Overall Population	*p*-Value
	(*N* = 25)	(*N* = 26)	(*N* = 51)	
Duration of surgery (min)	92.00 ± 38.19	93.46 ± 30.35		0.720
Duration of anesthesia (min)	121.60 ± 42.05	125.00 ± 33.55		0.597
Surgical approach OA TLA RPA	2 (8.0%) 15 (60.0%) 8 (32.0%)	6 (23.1%) 13 (50.0%) 7 (26.9%)	8 (15.7%) 28 (54.9%) 15 (29.4%)	0.334
Lowest intraoperative SBP (mmHg)	111.92 ± 10.08	88.65 ± 6.41		<0.001 *
Lowest intraoperative DBP (mmHg)	69.08 ± 8.70	52.31 ± 3.80		<0.001 *
Intraoperative crystalloid infusion (mL)	1500 (500–3000)	2000 (1000–3500)		<0.001 *
Intraoperative antihypertensive administration Nitrates Nitrates plus urapidil None	9 (36.0%) 5 (20.0%) 11 (44.0%)	8 (30.8%) 13 (50.0%) 5 (19.2%)	17 (33.3%) 18 (35.3%) 16 (31.4%)	0.054
Intraoperative vasopressor administration Yes No	0 (0.0%) 25 (100.0%)	13 (50.0%) 13 (50.0%)	13 (25.5%) 38 (74.5%)	<0.001 *
Intraoperative diuretic administration Yes No	3 (12.0%) 22 (88.0%)	1 (3.8%) 25 (96.2%)	4 (7.8%) 47 (92.2%)	0.279
Length of hospital stay (days)	3.28 ± 1.45	3.35 ± 1.52		0.906
Postoperative hypotension Yes No	9 (36.0%) 16 (64.0%)	10 (38.5%) 16 (61.5%)	19 (37.3%) 32 (62.7%)	0.856

* Statistically significant (*p* < 0.05). Data are shown as mean ± SD, median (range), or n (%). SBP—systolic blood pressure; DBP—diastolic blood pressure; OA—open adrenalectomy; TLA—transperitoneal laparoscopic adrenalectomy; RPA—retroperitoneoscopic adrenalectomy. Statistical comparisons: independent *t*-test or Mann–Whitney U-test for continuous variables, chi-square or Fisher’s exact test for categorical variables. *p* < 0.05 considered statistically significant.

For further statistical analyses and given the limited sample size, tumor diameter and intraoperative fluid administration were dichotomized based on their mean values. Tumors smaller than 49 mm were classified into one group, while tumors with a diameter ≥49 mm were classified into the other. Considering the duration of surgery, intraoperative fluid replacement was categorized into two groups: <1200 mL/h and ≥1200 mL/h. These dichotomized variables were subsequently included in a logistic regression model to evaluate their association with the occurrence of intraoperative hypotension.

Univariate logistic regression analysis (Table 4) identified several variables potentially associated with intraoperative hypotension. Tumor diameter was significantly associated with an increased risk of hypotension (OR 1.038, 95% CI 1.006–1.071, *p* = 0.018). A higher PASS score showed a trend toward increased risk, although it did not reach statistical significance (OR 1.344, 95% CI 0.970–1.863, *p* = 0.076). Among intraoperative factors, higher crystalloid infusion rate (OR 1.002, 95% CI 1.001–1.004, *p* = 0.002) and administration of antihypertensive agents (OR 2.399, 95% CI 1.146–5.020, *p* = 0.020) were significantly associated with hypotension. Other variables, including age, sex, tumor side, catecholamine and metabolite levels, preoperative blood pressure, type of alpha-blocker, surgical approach, duration of surgery or anesthesia, use of diuretics, length of hospital stay, and postoperative interventions, were not significantly associated with intraoperative hypotension.

Variables that reached a significance threshold of *p* < 0.1 in the univariate analysis were included in the multivariate logistic regression model to identify independent predictors of intraoperative hypotension. Based on the univariate results, the following variables were entered into the model: tumor diameter, PASS score, intraoperative crystalloid infusion rate, and administration of intraoperative antihypertensive agents. The multivariate analysis was performed using a backward stepwise approach, and model fit was assessed using the Nagelkerke R^2^ coefficient. Statistical significance for the multivariate model was set at *p* < 0.05.

Table 5 presents the results of the univariate and multivariate logistic regression analyses for predictors of intraoperative hypotension. In the multivariate model, only larger tumor size (diameter ≥ 49 mm; OR = 0.176, 95% CI 0.034–0.895, *p* = 0.036) and higher intraoperative crystalloid infusion (≥1200 mL/h; OR = 0.132, 95% CI 0.030–0.574, *p* = 0.007) remained independent predictors of intraoperative hypotension. PASS score and intraoperative antihypertensive administration were not significant predictors in the adjusted model. The multivariate logistic regression model was statistically significant (χ^2^ = 14.244, df = 4, *p* = 0.007). The Nagelkerke R^2^ was 0.331, indicating that 33.1% of the variance in intraoperative hypotension was explained by the included variables.

## 4. Discussion

Our study demonstrated that intraoperative hypotension occurred in 51% of patients. The most significant predictors of intraoperative hypotension were tumor size and the volume of fluids administered intraoperatively.

Numerous studies have identified tumor size as a key determinant of perioperative hemodynamic instability [12,22]. Adrenal pheochromocytomas synthesize, store, and release catecholamines, and larger tumors typically exhibit greater endocrine activity, predisposing patients to pronounced intraoperative hypertension. Conversely, resection of larger tumors is associated with chronic intravascular volume depletion and an abrupt decline in circulating catecholamine levels, mechanisms that may contribute to significant postoperative hypotension [23]. Moreover, surgical removal of larger tumors usually requires more extensive intraoperative manipulation, further increasing the risk of hemodynamic fluctuations.

Notably, the meta-analysis by Urabe et al. [11] demonstrated that tumor size significantly influences the development of intraoperative hemodynamic instability, contributing both to hypertensive episodes driven by excessive catecholamine secretion and to hypotensive events attributable to sudden catecholamine withdrawal following tumor resection [9]. In our study, multivariate logistic regression analysis confirmed that larger tumors (diameter ≥ 49 mm) were associated with an increased risk of intraoperative hypotension (*p* = 0.036; OR 0.176, 95% CI 0.034–0.895).

Over the past two decades, in addition to the transperitoneal laparoscopic approach (TLA), the posterior retroperitoneoscopic approach (PRA) has become widely established in adrenal surgery. Tumor size is frequently cited as a key factor influencing the surgeon’s choice between TLA and PRA [24]. Chen et al. [25] reported that PRA was independently associated with an increased risk of intraoperative hypotension, with multivariate analysis demonstrating a higher likelihood of mean arterial pressure <60 mmHg (OR 4.44). Similarly, Chaman Baz et al. [26] observed significantly higher intraoperative hypotension scores in PRA compared with TLA (97 vs. 46; *p* < 0.001), attributable to more frequent (IQR 2–5 vs. 1–3; *p* = 0.025) and longer hypotensive episodes, as well as prolonged bradycardic events. In our cohort, the surgical approach was not significantly associated with intraoperative hypotension (*p* = 0.334); however, open adrenalectomy was more frequently performed in patients with larger tumors, which may confound results since that tumor size is independently associated with hemodynamic instability. These findings highlight the importance of thorough preoperative evaluation and individualized intraoperative management for patients undergoing open adrenalectomy for large adrenal tumors to reduce the risk of intraoperative hypotension.

Walz et al. [27], who have not routinely pretreated pheochromocytoma patients with alpha-blockade since 2010, reported no increased risk of hypotension when comparing partial with total adrenalectomy. In their cohort, patients undergoing total adrenalectomy had larger tumors (4.5 ± 2.1 vs. 2.9 ± 1.2 cm; *p* < 0.001) and more frequently symptomatic disease (83% vs. 73%; *p* < 0.05), whereas partial adrenalectomy was associated with lower intraoperative peak systolic blood pressure [25].

In our study, nearly 10% of patients (5/51) did not receive preoperative alpha-adrenergic blockade. Two patients were unable to tolerate alpha-blockade, while in the remaining three patients a preoperative diagnosis of pheochromocytoma had not been established. Intraoperative hypotension was observed even in these patients despite the absence of alpha-blockade; however, due to the small sample size, this finding did not reach statistical significance. Nevertheless, current recommendations advocate preoperative alpha-adrenergic blockade even in patients with clinically silent pheochromocytoma [28].

In addition to tumor size, the volume of intraoperatively administered fluids emerged as a significant factor in our study. Patients who developed intraoperative hypotension received a mean crystalloid volume of 2068.27 ± 591.75 mL. In contrast, Pisarska-Adamczyk et al. [29] reported a mean intraoperative fluid replacement of 1595 ± 674.9 mL, which was not identified as a significant predictor of intraoperative hemodynamic instability. In that study, tumor size and the presence of diabetes mellitus were identified as independent predictors, unlike in our cohort.

Anesthetic management strategy also plays a crucial role in the development of hemodynamic instability. Decisions regarding fluid administration and subsequent initiation of vasopressor therapy are often based on individual clinical judgment, particularly in the absence of invasive hemodynamic monitoring, such as assessment of systemic vascular resistance. The use of epidural anesthesia has been shown to be a significant predictor of hemodynamic instability, especially sustained hypotension. The incidence of intraoperative hypotension in patients receiving epidural anesthesia has been reported to reach 72.9% [30]. Similarly, Jeon et al. [31] demonstrated that combined general and epidural anesthesia was not effective in preventing hypertensive crises and was associated with a higher incidence of intraoperative hypotension.

Despite thorough preoperative preparation, intraoperative hypotension remains a significant clinical challenge [32]. Data regarding the relationship between preoperative catecholamine levels and intraoperative hypotension are limited. In a single-center retrospective study involving 123 patients undergoing laparoscopic adrenalectomy for pheochromocytoma, nearly half developed prolonged intraoperative hypotension. These patients exhibited higher urinary catecholamine levels compared with those without prolonged hypotension, with preoperative urinary epinephrine and dopamine identified as the strongest predictors [33]. Another retrospective study reported greater intraoperative hemodynamic variability in patients with higher catecholamine levels [34].

In contrast to these findings, our analysis did not demonstrate a significant association between intraoperative hypotension and elevated preoperative catecholamine levels. This discrepancy may be explained by substantial data dispersion within our cohort, as reflected by a standard deviation approaching the mean catecholamine concentration, which may have limited statistical power. Nevertheless, the available evidence is largely constrained by retrospective study designs and heterogeneity in perioperative management strategies, underscoring the need for standardized prospective investigations.

Catecholamine levels may also influence postoperative hemodynamics. Weingarten et al. [34] reported that patients with more pronounced catecholamine excess were more likely to develop postoperative hypotension requiring vasopressor support. Similarly, a Japanese study involving more than 70 patients demonstrated a positive association between urinary epinephrine levels and postoperative hypotension requiring treatment [35]. Chang et al. [36] further showed that patients with perioperative hemodynamic instability had significantly higher urinary catecholamine levels, identifying norepinephrine as the strongest predictor, particularly in relation to postoperative hypotension.

Taken together, current evidence suggests that elevated preoperative catecholamine levels are associated with both intraoperative hemodynamic instability and postoperative hypotension in patients undergoing adrenalectomy for pheochromocytoma. While catecholamine profiling shows promise for perioperative risk stratification, robust prospective studies are required before its routine integration into clinical decision-making or perioperative protocols.

## 5. Conclusions

Several limitations should be considered in this study. The retrospective design and relatively small sample size should be acknowledged. Intraoperative hemodynamic data were sourced from manually recorded anesthesia charts, and the absence of an electronic anesthesia record system may have affected the accuracy and completeness of data collection, thereby limiting the precision of the analyzed hemodynamic parameters. Additionally, detailed preoperative cardiovascular assessments, imaging characteristics, genetic testing, and metabolic profiles were not consistently available, which may have limited the identification of all potential predictors of intraoperative hypotension.

Tumor size and intraoperative fluid administration emerged as independent predictors of intraoperative hypotension in patients undergoing surgical treatment for pheochromocytoma. These findings underscore the importance of careful preoperative risk stratification and individualized intraoperative hemodynamic management, particularly with regard to fluid therapy and timely initiation of vasopressor support. Future prospective studies with larger cohorts and standardized hemodynamic monitoring protocols are warranted to improve perioperative safety and outcomes in this high-risk patient population.

## Figures and Tables

**Table 1:** Characteristics of patients.

Characteristics	*N* (%) = 51
Sex Female Male	31 (60.8%) 20 (39.2%)
Age (years, mean ± SD)	53.08 ± 14.09
Tumor diameter (mm, mean ± SD)	49.04 ± 29.41 (13–144)
Smoking Smoker Non-Smoker	22 (42.2%) 29 (57.8%)
Side of PHEO Left-sided Right-sided	23 (45.1%) 28 (54.9%)
Surgical approach Open adrenalectomy Laparoscopic adrenalectomy Retroperitoneoscopic adrenalectomy	8 (15.7%) 28 (54.9%) 15 (29.4%)
Cateholamine levels and their metabolites	Reference range * Preoperative measurements
24 h urinary norepinephrine (nmol/24 h)	<570.0 2068.18 ± 3783.39
24 h urinary epinephrine (nmol/24 h)	<150.0 334.34 ± 488.97
24 h urinary dopamine (nmol/24 h)	<3240.0 2598.09 ± 2067.32
24 h urinary metanephrine (µmol/24 h)	<1.40 22.45 ± 50.93
24 h urinary normetanephrine (µmol/24 h)	<3.65 35.08 ± 97.09
Preoperative SBP (before alpha-blocker) (mmHg, mean ± SD)	181.25 ± 35.40
Preoperative DBP (before alpha-blocker) (mmHg, mean ± SD)	101.10 ± 20.07
Alpha blocker Phenoxybenzamine Doxazosin None	22 (43.1%) 24 (47.1%) 5 (9.8%)

Data are presented as mean ± SD for continuous variables and n (%) for categorical variables. PHEO, pheochromocytoma; SBP, systolic blood pressure; DBP, diastolic blood pressure. * Reference ranges are provided according to institutional laboratory standards.

**Table 4:** Univariate logistic regression analysis.

Variables	Odds Ratio (95%CI)	*p*-Value
Sex	0.938 (0.305–2.886)	0.910
Age	1.028 (0.987–1.072)	0.186
Smoking	0.771 (0.236–2.524)	0.668
Tumor diameter (mm)	1.038 (1.006–1.071)	0.018 *
Side of PHEO	1.259 (0.417–3.800)	0.683
PASS score	1.344 (0.970–1.863)	0.076
24 h urinary epinephrine (nmol/24 h)	1.000 (0.998–1.001)	0.548
24 h urinary norepinephrine (nmol/24 h)	1.000 (1.000–1.000)	0.928
24 h urinary dopamine (nmol/24 h)	1.000 (1.000–1.000)	0.387
24 urinary metanephrine (µmol/24 h)	1.002 (0.990–1.013)	0.782
24 urinary normetanephrine (µmol/24 h)	1.000 (0.994–1.006)	0.991
Preoperative SBP (mmHg) (before alpha-blocker)	1.011 (0.994–1.028)	0.201
Preoperative DBP (mmHg) (before alpha-blocker)	0.996 (0.969–1.024)	0.762
Preoperative Alpha blocker administration	1.371 (0.580–3.238)	0.472
Duration of surgery (min)	1.001 (0.985–1.018)	0.877
Duration of anesthesia (min)	1.002 (0.988–1.017)	0.745
Surgical approach	0.628 (0.292–1.350)	0.233
Intraoperative crystalloid infusion (mL/h)	1.002 (1.001–1.004)	0.002 *
Intraoperative antihypertensive administration	2.399 (1.146–5.020)	0.020 *
Intraoperative diuretic administration	0.293 (0.028–3.029)	0.303
Length of hospital stay (days)	1.031 (0.708–1.503)	0.872
Postoperative hypotension	1.111 (0.357–3.461)	0.856

Data are presented as odds ratio (OR) with 95% confidence intervals (CI). PHEO = pheochromocytoma; SBP = systolic blood pressure; DBP = diastolic blood pressure; mL/h = milliliters per hour. * Statistical significance was defined as *p* < 0.05.

**Table 5:** Univariate and multivariate logistic regression analysis to predict intraoperative hypotension.

	Univariate		Multivariate	
Variables	OR (95% CI)	*p*	OR	*p*
PASS score	1.344 (0.970–1.863)	0.076	0.893 (0.243–3.286)	0.865
Tumor diameter (mm)	1.038 (1.006–1.071)	0.018	0.176 (0.034–0.895)	0.036 *
Intraoperative cristalloid infusion (mL/h)	1.002 (1.001–1.004)	0.002	0.132 (0.030–0.574)	0.007 *
Intraoperative antihypertensive administration	2.399 (1.146–5.020)	0.020	0.736 (0.156–3.486)	0.700

PASS = Pheochromocytoma of the Adrenal Gland Scaled Score; OR = odds ratio; CI = confidence interval; mL/h = milliliters per hour; variables included in the multivariate model were selected based on a univariate significance threshold of *p* < 0.1. Tumor diameter was dichotomized at 49 mm, and intraoperative crystalloid infusion at 1200 mL/h. * Statistical significance was set at *p* < 0.05.

## Data Availability

The data presented in this study are available on request from the corresponding author due to privacy and ethical reasons.
